# Curbing the Influence of the Drug Industry: A British View

**DOI:** 10.1371/journal.pmed.0020241

**Published:** 2005-08-02

**Authors:** Smith Richard

## Abstract

Smith explores the implications of a recent report from Britain's House of Commons Health Committee that examined the pharmaceutical industry's influence upon medicine.

Britain's House of Commons Health Committee has recently recommended a fundamental realignment of the relationships between the pharmaceutical industry and government, regulators, doctors, the health service, and patients [1]. The committee said that the industry has interdigitated itself into every aspect of health care, and that government and others, including doctors, have taken the easy route of assuming that the interests of the industry and of the health services and patients are the same.

The committee's report makes clear that reducing the influence of the industry would be good for everybody, including—paradoxically—the industry itself, which could concentrate on developing new drugs rather than on corrupting doctors, patient organisations, and others. “It is not in the long term interests of the industry for prescribers and the public to lose faith in it,” says the report. “We need an industry which is led by the values of its scientists not those of its marketing force.”

## Select Committees: Rationality before Realpolitik

The Health Committee is one of many select committees of the House of Commons. The committees are comprised of members of parliament and politicians from all parties, and they can choose to examine any subject that raises matters of public importance. They receive written and oral evidence, including from government ministers, and produce reports and recommendations to which the government is required to respond.

The 11-member Health Committee chose to examine the influence of the drug industry because of increasing public concern that this influence is excessive. The committee was particularly worried by the industry's role in promoting “medicalisation,” the idea of a pill for every ill: “What has been described as the ‘medicalisation’ of society—the belief that every problem requires medical treatment—may also be attributed in part to the activities of the pharmaceutical industry” [1]. The committee, whose terms of reference are shown in Box 1, was also worried by the high prevalence of drug side effects. It heard from every interested party, including representatives of the drug companies, patients, doctors, medical journal editors, critics of the industry, and government ministers and officials.

**Box 1. Terms of Reference for the Health Committee Enquiry**

“The Health Committee is to undertake an inquiry into the influence of the pharmaceutical industry on health policies, health outcomes and future health priorities and needs. The inquiry will focus, in particular, on the impact of the industry on the following:

- drug innovation
- the conduct of medical research
- the provision of drug information and promotion
- professional and patient education
- regulatory review of drug safety and efficacy
- product evaluation, including assessments of value for money

In doing so, the Committee will examine the influence of the pharmaceutical industry on the NHS; National Institute for Clinical Excellence (NICE); regulatory authorities and advisory and consultative bodies; prescribers, suppliers and providers of medicines; professional, academic and educational institutions; the (professional and lay) press and other media; and patients, consumers, the general public and representative bodies.”

(Information taken from [1])

The government does not have to accept the recommendations from select committees, and it recently rebuffed recommendations from the same Health Committee encouraging open access to scientific research [2]. Usually, the committees will be much bolder than the government, which is heavily lobbied and pays more attention to realpolitik than to rational argument. Just as the publishing industry pressured the government to ignore recommendations on open access [3], the pharmaceutical industry will be doing the same now—and the industry is powerful; it is Britain's third most profitable economic activity (after tourism and finance) and employs 83,000 people.

## The All-Pervasive and Persistent Influence of the Industry

Although the pharmaceutical industry is now perceived by the public as putting profits ahead of patients' well-being [4], it is generally, as the committee makes clear, a force for good. Almost all of the drugs that have transformed medicine in the past half century have been developed and manufactured by the industry. “The discovery, development and effective use of drugs,” says the committee, “have improved many people's quality of life, reduced the need for surgical intervention and the length of time spent in hospital and saved many lives” [1]. And making the industry into a scapegoat for failing to produce drugs for the diseases of the poor is in some ways no more sensible, I believe, than blaming washing machine manufacturers for poor hygiene standards in the developing world. The industry is part of the for-profit sector, and has what many philosophers might call a moral duty to maximise profits. Producing drugs for the poor requires imaginative public–private partnerships.

It's also shallow thinking to view the industry as corrupters and doctors as the corrupted. As a doctor myself, I think that doctors are in many ways to blame for the debased relationship between themselves and the industry. The industry is (mostly) behaving in ways that are “normal” within the commercial sector. It is the doctors who depart from their ethical base when they insist on first-class fares and lavish entertainment from the industry so that they can attend an international conference.

The fundamental problem, says the committee, is that the pharmaceutical industry's influence is too pervasive: “The industry affects every level of healthcare provision, from the drugs that are initially discovered and developed through clinical trials, to the promotion of drugs to the prescriber and the patient groups, to the prescription of medicines and the compilation of clinical guidelines.”

The committee goes into detail about each of these levels. Regulatory authorities, it says, are too close to the industry, meaning that they do not ensure that the industry works in the public interest. The clinical trials that are the essential evidence base for regulatory and clinical decisions are produced almost entirely by the industry, and the evidence that reaches authorities, doctors, and patients is biased. Guidelines for treating patients are distorted, not only because they must be based on biased evidence, but also because the organisations and people producing them are often in hock to the industry. The organisations may receive millions of British pounds for buildings and activities, while the individuals—particularly key opinion leaders (KOLs, as they are known in the trade)—may receive hundreds of thousands of pounds for consultancy, speaking fees, travel, research, and articles. “Drug companies are criticised for giving hospitality and recruiting ‘key opinion leaders’,” says the committee, “but the prescribers must be equally to blame for accepting the hospitality and some ‘key opinion leaders’ for lending their names to work they did not produce, often for very considerable sums.”

Next in the list of things that concerned the committee comes the industry's intensive marketing, which is becoming ever more important as the flow of drugs that offer major therapeutic advances (and so need much less marketing) dries up. Britain has some 8 000 drug company representatives, but the industry also spends millions on advertising, sponsorship, meetings, and increasingly, “medical education”—which often means a fine dinner and a lecture from a captive KOL. The report states: “Coupled with company-sponsored information from medical journals and supplements, ‘medical education’ materials, advertisements and sponsorship to attend conferences, workshops and other events, it is little wonder that prescribing practices are affected.” Medical journals, as I've argued in *PLoS Medicine* [3], are in some ways extensions of the marketing arm of the industry, while the free newspapers that overwhelm doctors in the developed world depend 100% on largesse from the industry.

Individual journalists are also captured, the committee heard—and perhaps most troublesome is the way patient organisations have become so dependent on the industry. The committee concluded that “Measures to limit the influence of industry on patient groups are needed.” Currently, in Britain, we see that the “patients” who are trying to convince the British government that it should ignore the advice of the London-based National Institute of Health and Clinical Excellence (NICE) (which says that drugs for Alzheimer's disease are not sufficiently cost effective) are in many ways agents of the companies that produce those drugs [6].

The consequences of all of these incestuous relationships, says the committee, are bad decisions on the regulation and prescription of drugs, over-reliance on drugs rather than on other interventions (such as dietary change, exercise, or counselling), and the “medicalisation” of life's problems, including baldness, shyness, unhappiness, grief, and sexual difficulties.

## Recommendations: “Let the Sun Shine In”

The committee came up with 48 conclusions and recommendations, and I have listed some of the highlights in Box 2. The committee's main recommendation for the problems it identifies is transparency: “let the sun shine in.” It begins by recommending that there be a clinical trials register, “maintained by an independent body” and containing full information. Companies should be required to put the information on the register “at launch as a condition of the marketing licence.” The committee also wants regulatory authorities and ethics committees (the British equivalent of institutional review boards) to help with the design of trials to make sure that they are answering real questions. It didn't, however, recommend more public funding of trials. I believe that such funding is necessary in order to ensure that trials are addressing the most important questions—including head-to-head comparisons and trials of new drugs against older drugs and non-drug treatments. Advice to companies is unlikely to be effective.

**Box 2. Recommendations from the Health Committee Enquiry: Some Highlights**

- The process of licensing drugs, and the medicines' regulatory system, should both be more transparent.
- There should be an independent register of clinical trials.
- Clinical trials should focus on using health outcomes that are relevant to patients.
- More research should be undertaken into the adverse effects of drugs and the costs of drug-induced illness.
- The regulator should ensure greater restraint in medicines' promotion.
- Tougher restriction should be placed on the prescribing activities of non-specialists.
- Doctors should be required to declare significant sums or gifts they receive as hospitality.
- The sponsorship of the drug industry should pass from the Department of Health to the Department of Trade and Industry—because the secretary of state for health cannot serve two masters (the public and the industry).
- (Information taken from [1])

There should be, says the committee, limits on the quantity of marketing materials, particularly in the first six months after launch, and stricter controls on marketing to junior doctors, nurses, and pharmacists. These proposals don't seem sufficiently thought through: it's hard to imagine how the proposals would be enforced, and they are patronising to junior doctors, nurses, and pharmacists—many of whom are much better, I suspect, at assessing evidence than burnt out, ageing, high-prescribing general practitioners.

The Health Committee would also like to see an independent review of the Medicines and Healthcare Products Regulatory Agency (London, United Kingdom) plus a public inquiry every time a drug is withdrawn from the market on health grounds. It's hard to see the government implementing these recommendations, as inquiries are expensive and always create difficulties for government, but if bodies like the Medicines and Healthcare Products Regulatory Agency and NICE are to maintain public confidence they will have to distance themselves from the industry—and be seen to do so. Important first steps will be to make public more of the information they use to make their decisions and to exclude KOLs from their committees (which may be difficult, as KOLs include many prominent doctors and professors of pharmacology and therapeutics).

Doctors' organisations, says the committee, should produce publicly available registers of doctors' links with industry. These registers—and this is my recommendation and not the committee's—should also include information on monetary amounts. Otherwise, it will not be possible to separate the KOLs from the vast numbers of doctors who receive pens, lunches, trips, and other gifts from the pharmaceutical industry. I doubt very much that doctors' organisations will adopt these recommendations until forced to do so. In Britain, it's more embarrassing to ask people about money than sex. Plus, doctors might come to be seen as the villains rather than the good guys.

The committee also wants patients' organisations to declare their connections with industry and to make clear when ubiquitous “disease awareness” campaigns are funded by the industry, which is probably very common [6]. I agree with this support for transparency, and while recognising the penury of many patients' organisations, I think they would do well to resist the lure of the industry's lucre as much as they can.

## Conclusion

In the end, this report will probably be less remembered for its recommendations—most of which will probably be ignored—than for having brought the important debate over the excessive influence of the pharmaceutical industry to a broader public. We all stand to benefit from the reduction of that influence.

## Abbreviations

KOL: key opinion leader
NICE: National Institute of Health and Clinical Excellence
